# Retinal peri-arteriolar versus peri-venular amyloidosis, hippocampal atrophy, and cognitive impairment: exploratory trial

**DOI:** 10.1186/s40478-024-01810-2

**Published:** 2024-06-28

**Authors:** Oana M. Dumitrascu, Jonah Doustar, Dieu-Trang Fuchs, Yosef Koronyo, Dale S. Sherman, Michelle Shizu Miller, Kenneth O. Johnson, Roxana O. Carare, Steven R. Verdooner, Patrick D. Lyden, Julie A. Schneider, Keith L. Black, Maya Koronyo-Hamaoui

**Affiliations:** 1 Departments of Neurology, Mayo Clinic, AZ, 13400 E. Shea Blvd, Scottsdale, AZ 85259 USA; 2 Department of Neurosurgery, Cedars-Sinai Medical Center, Maxine Dunitz Neurosurgical Institute, 127 S. San Vicente Blvd., Los Angeles, CA 90048 USA; 3 Department of Physical Medicine and Rehabilitation, Cedars-Sinai Medical Center, 127 S. San Vicente Blvd., Los Angeles, CA 90048 USA; 4 Department of Neurosurgery, Tulane University School of Medicine, 1415 Tulane Ave, New Orleans, LA 70112 USA; 5 NeuroVision Imaging LLC, 1395 Garden Hwy, Sacramento, CA 95833 USA; 6 Department of Clinical Neuroanatomy, University of Southampton, University Road Southampton, Southampton, SO17 1BJ UK; 7 Department of Physiology and Neuroscience, Zilkha Neurogenetic Institute, Keck School of Medicine, University of Southern California, 1501 San Pablo St, Los Angeles, CA 90033 USA; 8 Department of Pathology, Department of Neurological Sciences, Alzheimer’s Disease Research Center, Rush Medical College, Rush University, 600 S. Paulina St., Chicago, IL 60612 USA; 9 Department of Neurology, Cedars-Sinai Medical Center, 127 S. San Vicente Blvd., Los Angeles, CA 90048 USA; 10 Division of Applied Cell Biology and Physiology, Department of Biomedical Sciences, Cedars-Sinai Medical Center, 127 S. San Vicente Blvd., Los Angeles, CA 90048 USA

**Keywords:** Perivascular, Amyloid imaging, Retina, Neurodegeneration, Cognition, Hippocampal volume, Peri-arteriolar, Peri-venular

## Abstract

The relationship between amyloidosis and vasculature in cognitive impairment and Alzheimer’s disease (AD) pathogenesis is increasingly acknowledged. We conducted a quantitative and topographic assessment of retinal perivascular amyloid plaque (AP) distribution in individuals with both normal and impaired cognition. Using a retrospective dataset of scanning laser ophthalmoscopy fluorescence images from twenty-eight subjects with varying cognitive states, we developed a novel image processing method to examine retinal peri-arteriolar and peri-venular curcumin-positive AP burden. We further correlated retinal perivascular amyloidosis with neuroimaging measures and neurocognitive scores. Our study unveiled that peri-arteriolar AP counts surpassed peri-venular counts throughout the entire cohort (*P* < 0.0001), irrespective of the primary, secondary, or tertiary vascular branch location, with a notable increase among cognitively impaired individuals. Moreover, secondary branch peri-venular AP count was elevated in the cognitively impaired (*P* < 0.01). Significantly, peri-venular AP count, particularly in secondary and tertiary venules, exhibited a strong correlation with clinical dementia rating, Montreal cognitive assessment score, hippocampal volume, and white matter hyperintensity count. In conclusion, our exploratory analysis detected greater peri-arteriolar versus peri-venular amyloidosis and a marked elevation of amyloid deposition in secondary branch peri-venular regions among cognitively impaired subjects. These findings underscore the potential feasibility of retinal perivascular amyloid imaging in predicting cognitive decline and AD progression. Larger longitudinal studies encompassing diverse populations and AD-biomarker confirmation are warranted to delineate the temporal-spatial dynamics of retinal perivascular amyloid deposition in cognitive impairment and the AD continuum.

**Supplementary Information:**

The online version contains supplementary material available at 10.1186/s40478-024-01810-2.

## Background

The vascular contribution to cognitive impairment and Alzheimer’s disease (AD) is increasingly being recognized [1]. Multiple studies have emphasized the role of various vascular derangements in neurodegeneration [2–10], with disruption of the blood–brain barrier proposed as an early biomarker of cognitive dysfunction in AD [1]. Given the similar pathophysiology shared between the blood-retina barrier and the blood–brain barrier [11–14], and the feasibility of non-invasive and reproducible high-resolution imaging of the retina, multiple static and dynamic retinal vascular biomarkers were investigated across the AD spectrum [11, 15–19]. Retinal vascular pathology in AD was characterized using techniques such as color and autofluorescence fundus photography, optical coherence tomography angiography [20–22], fluorescein angiography [23, 24] in humans, and retinal pericyte imaging in animal models [25]. Fundus photography has revealed several vascular abnormalities in AD, including venular narrowing, diminished vascular branching, increased tortuosity, and decreased arterial fractal dimension [19, 26–29], leading to the recent development of retinal photography-based deep learning algorithms for cost-effective AD screening in community settings [30, 31]. Furthermore, it has been proposed that retinal vascular tortuosity could improve the detection of cerebral amyloid status as determined by 18F-florbetaben PET [32]. Amyloid β-protein (Aβ) is an early core biomarker of AD, a prerequisite for AD diagnosis, and is the target of AD-specific therapies [33], including the recently approved anti-Aβ monoclonal antibodies that bind with high affinity to Aβ plaque and/or soluble protofibrils [34, 35]. These immune-based therapies have demonstrated efficacy in patients with mild cognitive impairment (MCI) and mild AD dementia [34–37].

Accurately detecting vascular-associated amyloid deposition in early AD remains an unmet need in current clinical practice. The cost-effective and non-invasive detection of retinal amyloid burden carries the potential for early AD identification and monitoring [2, 38–40]. Thus, several retinal amyloid imaging methodologies have been recently developed [41, 42], including scanning laser ophthalmoscopy (SLO) [17, 39, 42–46] and hyperspectral retinal imaging [13, 39–41]. SLO fluorescence imaging following curcumin administration provides an opportunity to visualize and quantify not only the Aβ plaques (AP) but also the retinal arteries and veins [17, 44, 45, 47]. We have previously reported the specificity of curcumin for retinal Aβ (especially for AD-linked Aβ_42_ alloforms) and its optical signature when bound to retinal Aβ deposits, both ex vivo and in vivo [41, 42]. Additionally, we have demonstrated that retinal venular tortuosity, combined with retinal mid-periphery AP count, can discriminate between patients with normal and impaired cognition [17]. Moreover, our group has identified significant accumulation of vascular and perivascular Aβ deposition in postmortem retinas of MCI (due to AD) and AD patients [16, 42, 48]. We further showed that increased histopathological arteriolar Aβ_40_ deposition and tight junction loss in the inner blood-retinal barrier of prodromal and symptomatic AD patients are strongly correlated with the severity of cerebral amyloid angiopathy (CAA) and other AD-related brain neuropathological changes [49].

While various vascular alterations have been described in retinal imaging studies in AD, the specific AP localization in the vascular-adjacent zones (termed here as perivascular AP) during preclinical or minimally symptomatic AD stages, and how these evolve with AD progression, remain unclear. To elucidate the retinal perivascular AP deposition in early AD, we conducted a retrospective exploratory analysis of a cohort of subjects with normal or impaired cognition who underwent retinal curcumin fluorescence amyloid imaging with SLO. Our aim was to study the topographic relationship between perivascular AP burden and retinal vessel types, determining whether retinal AP distribution is predominantly peri-arteriolar or peri-venular, proximal to the first-order vessels or further distal following secondary or tertiary vessel bifurcation. We hypothesized that an increased perivascular retinal AP burden would correlate with cognitive decline, and similar to the AD brain, retinal AP deposits would be more abundant in the peri-arteriolar space than in the peri-venular space. We compared the perivascular AP distribution in subjects with and without cognitive impairment and assessed the correlation between retinal peri-arteriolar and peri-venular AP counts with neuroimaging markers of neurodegeneration.

## Methods

### Study design

**Fig. 1:**
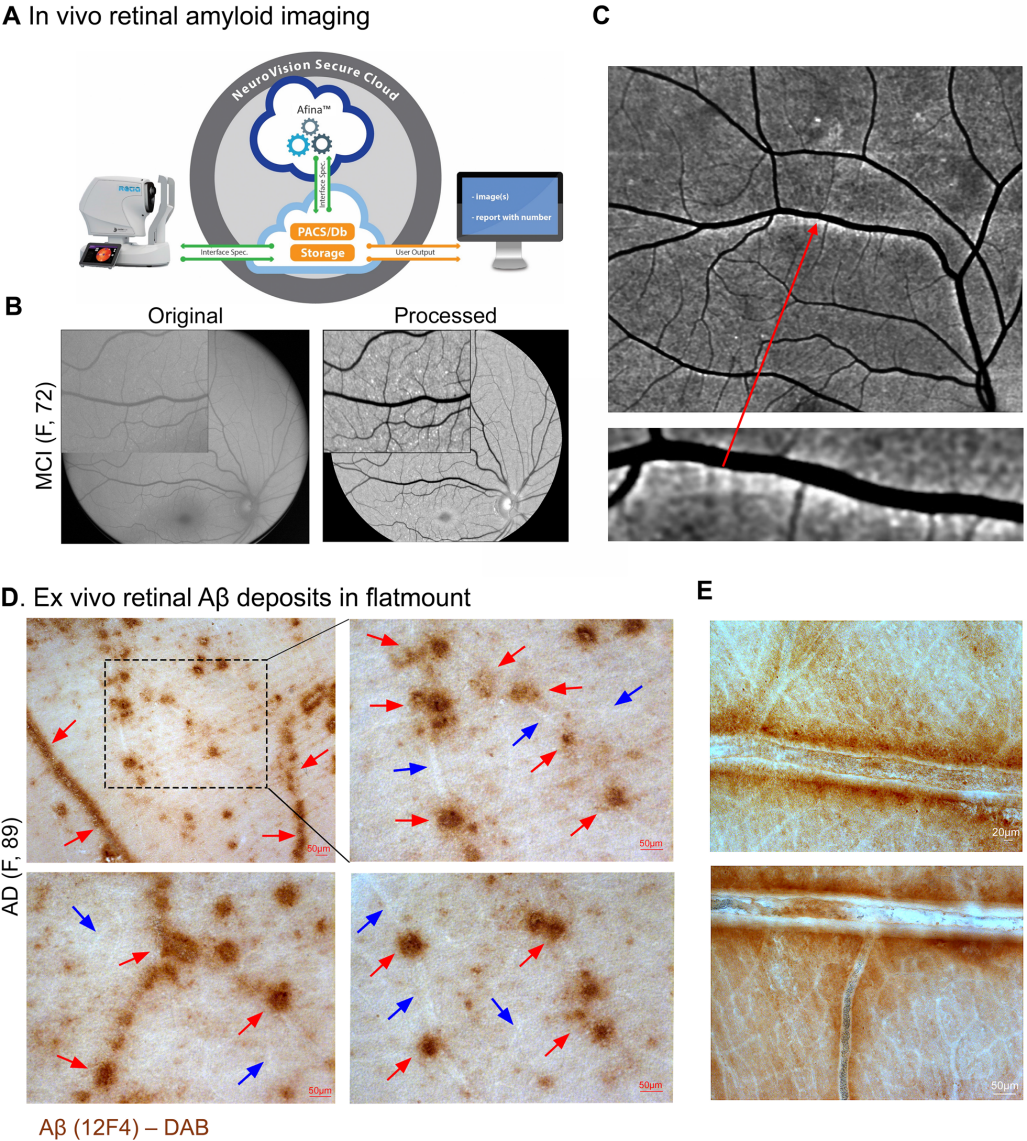
Retinal vascular amyloid imaging. **A** Pipeline of fluorescent imaging using Retia® SLO, Afina™ cloud storage, and a fully automated image processing and analyses. **B** Representative retinal fluorescent image of before and after image processing. **C** Representative retinal image of an AD patient demonstrating putative retinal amyloid deposits (white) along the blood vessels. **D** Representative histopathological images from a confirmed AD patient, that did not undergo retinal imaging pre-mortem. Postmortem retinal flatmount immunolabeled with anti-c monoclonal antibody (12F4; brown) and peroxidase-base 3,3′ diaminobenzidine (DAB) immunostaining. Typical Aβ plaque structures and Aβ deposition along and inside blood vessels (red arrows) and ‘plaque-free’ regions of retinal blood vessels (blue arrows). **E** High magnification images of perivascular and vascular Aβ deposits in retinal flatmounts of an AD patient that did not undergo retinal imaging pre-mortem

This is a retrospective retinal imaging investigation of a cohort study approved by the Cedars-Sinai Medical Center Ethics Committee and Institutional Review Board (IRB protocol number 00052349), which was conducted in accordance with the ethical standards outlined in the 1964 Declaration of Helsinki and its subsequent amendments or comparable ethical standards. The prospective parent study enrolled 34 subjects aged over 40, with subjective cognitive decline, that provided written informed consent prior to enrollment. All subjects underwent retinal imaging with a confocal scanning laser ophthalmoscope (SLO Retia™, CenterVue SpA; Fig. 1A), utilizing blue light to excite curcumin emission for obtaining fluorescent images of the retina (Fig. 1B), following a previously reported study design [17, 42, 44]. In addition, patients underwent brain magnetic resonance imaging (MRI), fludeoxyglucose-18 positron emission tomography (PET) and a comprehensive neuropsychometric evaluation (detailed below). Exclusion criteria for the parent retinal imaging study included a self-reported history of glaucoma (to avoid ocular dilation-triggered angle-closure), allergy to mydriatic eye drops, curcumin, or vitamin E. In the current retrospective study, we excluded subjects diagnosed with other neurodegenerative disorders (e.g. frontotemporal dementia) and subject with poor retinal image quality. Overall, this study included 28 subjects, with either normal cognition and normal neuroimaging (9), MCI (16; 6 amnestic MCI, 9 multidomain MCI, 1 non-amnestic MCI), or probable AD (3).

Standard neuropsychological testing was administered by a licensed neuropsychologist (DS) and included the Montreal Cognitive Assessment (MOCA), global Clinical Dementia Rating (CDR), general cognitive (ACS-test of Premorbid Functioning), and specific cognitive domain assessments: attention and concentration (Wechsler Adult Intelligence Scale (WAIS)-IV); verbal memory (California Verbal Learning Test (CVLT) II, Wechsler Memory Scale (WMS)-IV, and Logical Memory II); non-verbal memory (Rey Complex Figure Test and Recall (RCFT) 30 min, and Brief Visuo-Spatial Memory Test Revised (BVMT-R) Delayed Recall); language [Fluency-Letter (FAS) and Fluency-category (animals)]; visuo-spatial ability (Rey Complex Figure Test and Recognition Trial (RCFT) Copy); speed of information processing (Trails A and B); and symptom validity and functional status (SF-36 Physical Component Score (PCS) and Mental Component Score (MCS)). The subject’s emotional status was assessed using the Beck Depression Inventory II, Geriatric Depression Scale, and Profile of Mood State/Total Mood Disturbance. All subjects underwent 3 Tesla non-contrast structural MRI. Brain volumetric analysis was conducted using automated NeuroQuant software [50], and the following parameters were collected: total intracranial volume (ICV) (cm^3^), hippocampal volume (HV) (cm^3^), and inferior lateral ventricle volume (ILVV) (cm^3^). The number and volume of white matter hyperintensities (WMHI) were measured using SPIN Software (SpinTech, Bingham Farms, MI).

### Post-mortem human retinal flatmounts preparation and Aβ immunostaining

Two eyes from a deceased AD female patient, provided by the Rush University’s Alzheimer’s Disease Research Center (ADRC ORA# 18011111), were collected within 8 h postmortem. Eyes were punctured once at the limbus and then preserved in Optisol-GS media (Bausch & Lomb, 50006-OPT, Sterile) before storage at 4 °C for up to 24 h. Retinas were isolated from whole eyes and the vitreous was removed before flatmount preparation. The retinas then underwent antigen retrieval followed by immunostaining using anti-Aβ_42_ antibodies (Abs) and peroxidase-base 3,3’ diaminobenzidine (DAB) labeling, according to a previously described method [13, 42, 51]. In detail, fresh eyes were dissected over ice, with removal of the cornea, anterior/posterior chamber, pupil, and lens to create the eyecup. The vitreous body was thoroughly removed manually throughout this process. Eyecups were then washed with 1X PBS solution and fixed in 2.5% paraformaldehyde (PFA). Retinas were dissected, separated from the choroid, and prepared into flatmounts by dividing them into four quadrants (superior, temporal, inferior, and nasal). Next, retinal flatmounts were washed in 1X PBS then treated with target retrieval solution at 97 °C for 1 h (pH 6.1; Dako #S1699) and washed one more time in 1X PBS. The tissues were next treated with 3% hydrogen peroxide (H_2_O_2_) for 12 min and washed in 1X PBS. Thereafter, the tissues were immunostained using a Vectastain Elite ABC HRP kit (Vector Laboratories, USA #PK-6102, Peroxidase Mouse IgG), a sensitive avidin/biotin-based peroxidase enzyme system, according to the manufacturer’s instructions. Briefly, tissues were first treated with blocking solution and permeabilized with 0.2% Triton® X-100 (Sigma #T8787) for 30 min at room temperature. They were then incubated with primary mouse anti-human Aβ_42_ monoclonal Abs (clone 12F4; recognizing and specific for the 42aa C-terminus of Aβ; 1:500 in PBS with 10% permeabilization/blocking solution; Biolegend #805501). Slides were covered with parafilm and incubated for 48 h at 4 °C. Following incubation, tissues were washed with 1X PBS, followed by incubation with a biotinylated secondary antibody for 30 min, and then ABC reagent incubation for another 30 min. Aβ_42_ immunoreactivity was detected using the DAB plus substrate chromogen system (Dako #K3467). Afterward, tissues were mounted with Paramount aqueous mounting medium (Dako #S3025). Routine controls were processed using an identical protocol, omitting the primary antibody to assess nonspecific labeling. Bright-field images were acquired using a Carl Zeiss Axio Imager Z1 fluorescence microscope (Carl Zeiss MicroImaging, Inc.) equipped with ApoTome, AxioCam MRm, and AxioCam HRc cameras (Fig. 1D–E; Supplementary Fig. 1). Histological studies were performed under the IRB protocol Pro00055802 at Cedars-Sinai Medical Center.

### Retinal image processing and analysis

**Fig. 2:**
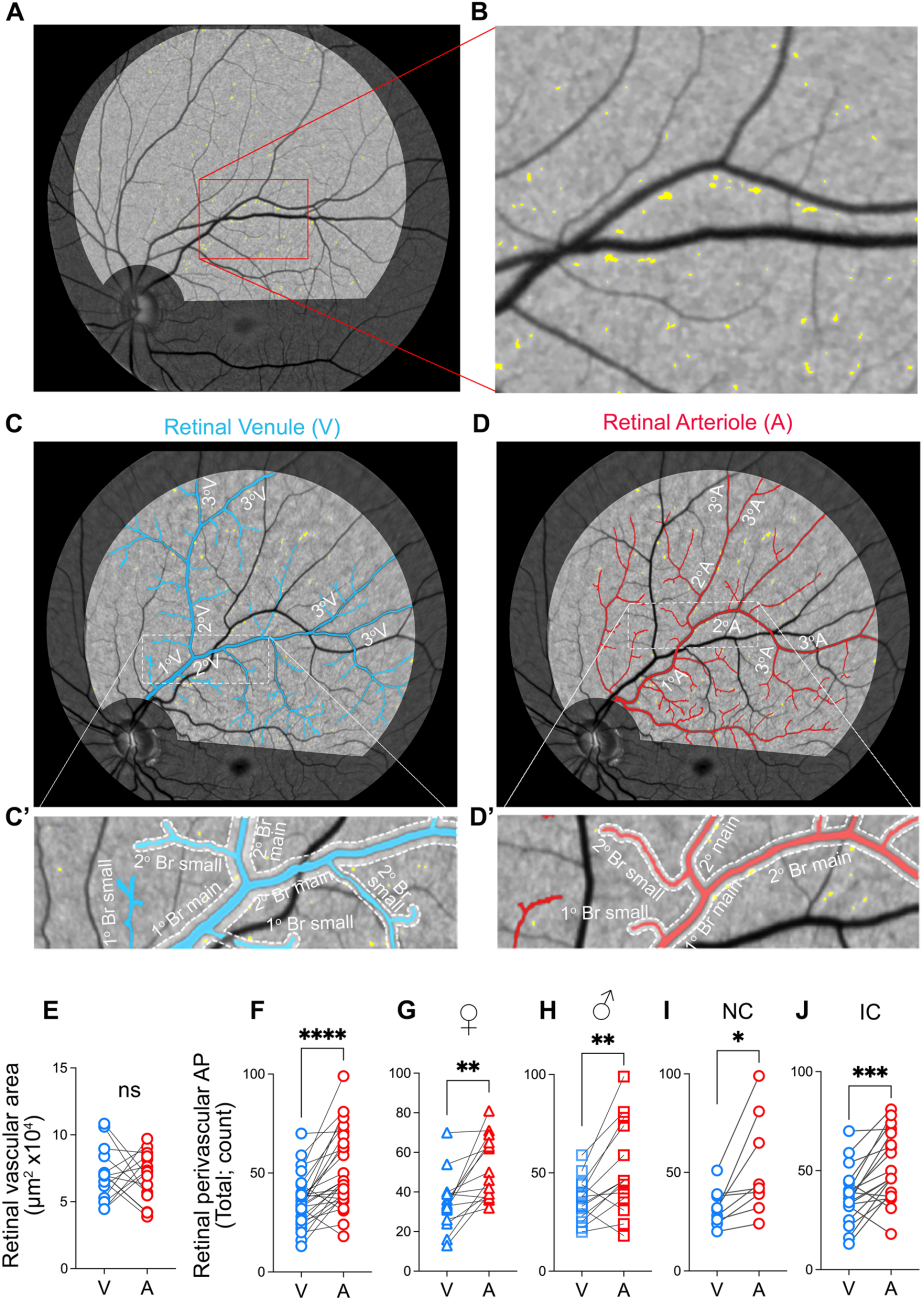
Retinal peri-venular and peri-arteriolar amyloid plaque distribution. **A**–**B** Representative retinal fluorescent fundus image illustrating retinal curcumin-positive amyloid hyperfluorescent plaques in the left eye supero-temporal quadrant (**A**, magnification **B**). Illustration of the primary, secondary, and tertiary retinal venular (**C**) and arteriolar (**D**) branches. Magnifications of the peri-venular (**C’**) and peri-arteriolar (**D’**) area used for the amyloid plaque (AP) quantification; boundary zone delineated by dotted lines producing a perivascular area of one equivalent vessel diameter on either side. **E** Quantitative analysis of retinal perivascular area stratified by venules (V) and arterioles (A) showing no significant difference between the total area for each vessel type in the analyzed supero-temporal region. **F**–**J** Quantitative analyses of retinal perivascular AP count stratified by V versus A in the total branches (**F**), in females (**G**), males (**H**), individuals with normal cognition (**I**) and impaired cognition (**J**). Individual data points are shown. * *P* < 0.05, ** *P* < 0.01, *** *P* < 0.001, **** *P* < 0.0001 by paired two-tailed Student’s t test. NC, Normal cognition; IC, Impaired cognition; 1°V, primary venular branch; 2°V, secondary venular branch; 3°V tertiary venular branch; 1°A, primary arteriolar branch, 2°A, secondary arteriolar branch; 3°A, tertiary arteriolar branches. Color code vessel type: blue—peri-venular; red—peri-arteriolar

The retinal AP imaging analysis was conducted on the left and right eyes following our previously reported methodology in a predominantly Caucasian population [44] which has recently been replicated in a Japanese population [45] and the A4 trial [39]. Noninvasive retinal amyloid images were acquired in predetermined geometrical regions using the Retia™ SLO, followed by fully automated Afina™-based cloud storage and NeuroVision imaging-based image processing output (Fig. 1A). The researchers responsible for the retinal image processing and AP quantification were blinded to the patients’ clinical characteristics. Retinal curcumin fluorescence imaging revealed multiple diffuse retinal hyperfluorescence spots (APs), which became more discernable after image processing (Fig. 1B, white dots). Quantification of perivascular AP (Fig. 1C) was performed in retinal fluorescence images obtained from the supero-temporal quadrant of the left eye. To achieve this, retinal arteries and veins were manually identified by trained observers based on their vessel diameter (vein > artery [52, 53]), location, and morphology, and traced using Adobe Photoshop CC 2020. Vessels originating from the optic disc were defined as the "primary (1°) main branch" for each venular and arteriolar network. After the defined main primary branch bifurcation, subsequent divisions were labeled as "secondary (2°) main branches" for all venules and arterioles. Following these main secondary arterioles and venules split, the subsequent divisions were termed "tertiary (3°) branches" (Fig. 2C–D). Any protruding vessels from the main branches were categorized as 'small.' For example, a protruding vessel from the main primary branch was labeled "primary branch—small". Similarly, if they protruded from the main secondary branch, they were denoted as "secondary branch—small" (Fig. 2C’–D’). Since tertiary vessels and their branches had relatively equal diameters, all vessels following the second bifurcation were grouped as tertiary branches. For analytical purposes, main branches along with protruding vessels were combined into one measurement, referred to as primary branches and secondary branches, encompassing both main and small primary and secondary branches, respectively.

Starting within the highlighted imaging field and nearest to the optic disc, we measured the diameter of the primary vessel at pre-set intervals along its length using the line segment tool. The diameter of each vessel was calculated by averaging these measurements from the pre-set intervals, as well as the most proximal and distal segments. Afterwards, we manually traced through the middle of the vessel using the curvature tool, prioritizing accuracy. The diameter of the drawn external perivascular line was set to automatically display three times the diameter of the vessel, producing a “perivascular area” equivalent to one vessel diameter on either side. We adjusted the line opacity to 40% to enable the detection of plaque positive signals within this perivascular area. Plaques that touched the border were counted as perivascular, even if their entire body was not within the boundary. These steps were repeated for all venules and arterioles to delineate all peri-arteriolar and peri-venular areas. We counted all hyperfluorescent AP positive signals falling within the boundaries of the perivascular areas, categorizing them by (1) vessel type (peri-venular versus peri-arteriolar), and (2) location (primary vessel, primary branch, secondary vessel, secondary branch, or tertiary vessel). Retinal images were independently analyzed by two graders (JD and MSM) to ensure reliability. Any discrepancies were discussed with a senior grader (OMD, YK, or MKH) for final clarification.

### Statistical analysis

Descriptive statistics were performed, and continuous demographic and clinical data are presented as the mean ± standard deviation (SD) in the text and tables. Comparisons between peri-arteriolar and peri-venular AP counts utilized paired and unpaired, two-tailed tests (unadjusted P) or one-way analysis of variance (ANOVA), corrected P values after the Tukey’s multiple comparisons posttest. Subjects were divided into three groups according to the Clinical Dementia Rating (CDR) (0.5, questionable impairment; 1, mild cognitive impairment; and 2, moderate cognitive impairment) [54]. They were also dichotomized using a MOCA cut-off of 26 [55] and neuropsychometric diagnosis (normal cognition versus impaired cognitive performance).

D'Agostino-Pearson and Shapiro–Wilk normality tests were conducted to assess whether continuous variables followed the normal distribution curve. Data set that passed at least one of the tests was considered to have a Gaussian distribution (Supplementary Table 1). Normally distributed variables were compared using two-tailed paired or unpaired Student’s *t*-test. For non-normally distributed independent variables, we utilized the two-tailed unpaired Mann–Whitney test. Fold changes (FC) and corresponding 95% confidence intervals (CI) were calculated (Tables 2 and 3). Differences in continuous variables among levels of CDR were examined through ANOVA with Tukey’s test applied to correct for multiple comparisons (Supplementary Table 4). Pearson’s *r* correlation analysis was conducted to investigate the relationship between retinal perivascular AP count and cognitive and brain imaging volumetric measures. The scatterplot graphs present the null hypothesis of a pair-wise Pearson’s *r* with the unadjusted P values that indicate the direction and strength of the linear relationship between two variables. Pearson’s correlations that remained significant after Holms-Bonferroni multiple comparison correction were indicated in Tables 4 and 5. All statistical analyses were performed using GraphPad Prism 9 or 10. Significance was defined as a P-value less than 0.05.

## Results

A total of 34 patients underwent retinal and brain imaging, as well as neuropsychometric evaluation. Figure 1C depicts a human retinal curcumin fluorescence imaging revealing APs putatively located along retinal blood vessels. Additionally, a representative microscopic image of Aβ immunohistochemical analysis in postmortem retinal flatmounts from an 89-year-old female with AD is provided to demonstrate multiple abluminal and perivascular APs accumulating along and inside retinal blood vessels (Fig. 1D-E).

We compared total temporal AP counts between the left and the right eye and observed a very strong and positive inter-eye correlation (r = 0.82, *P* = 0.0002; Supplementary Fig. 2). Since no difference between the left and right retinal AP counts was noted, we have arbitrarily chosen to conduct further analyses of AP counts within the supero-temporal quadrant of the left eyes. This is also based on prior retinal pathological and amyloid imaging studies highlighting that the supero-temporal quadrant has the highest burden of retinal AP, which is also most predictive of cognitive loss and hippocampal atrophy [16, 42, 44, 48, 56].

Perivascular AP analyses included twenty-eight patients who had suitable left eye supero-temporal retinal images for vascular tracing and perivascular AP quantitative analysis. The mean (SD) age was 65 (7.4) years, with 50% being female. The mean (SD) MOCA score was 25 (5.6), with a median MOCA score of 27 (range 4–32). Among the subjects, 11 had a CDR of 0.5, 14 had a CDR of 1, and 3 had a CDR of 2. Based on formal neuropsychometric cognitive evaluation corroborated with neuroimaging findings, 9 (32.1%) patients had normal cognition (NC), while 19 (67.9%) had impaired cognition (IC; 6 amnestic MCI, 9 multidomain MCI, 1 non-amnestic MCI, and 3 probable AD). The cohorts with normal and impaired cognition were matched for age, sex, and years of education (Table 1).

**Table 1:** Cohort demographic and cognitive characteristics

Demographics	All (n = 28)	Normal cognition (n = 9)	Impaired cognition (n = 19)	*P* value
Average age (SD)	65 (7.4)	65 (5.3)	65 (8.3)	0.83
Sex (male n (%))	14 (50%)	4 (44.4%)	10 (52.6%)	–
Years of Education Mean (SD)	17 (1.8)	17 (1.5)	17 (1.9)	0.94

Neurocognitive characterization	All (n = 28)	MOCA > 26 (n = 15)	MOCA ≤ 26 (n = 13)	
Average age (SD)	65 (7.4)	64 (7.2)	66 (7.6)	0.36
Sex (male n (%))	14 (50%)	6 (40%)	8 (60%)	–
Years of Education Mean (SD)	17 (1.8)	17 (1.5)	17 (2.1)	0.69

	Score	Total	Female	Male
Standard Global CDR (n = 28)	0.5	11	7	4 (36.4%)
	1	14	6	8 (57.1%)
	2	3	1	2 (66.7%)

Statistical analysis was established using two-tailed unpaired Student’s t test

*NC* normal cognition, *IC* impaired cognition (refers to patients with diagnosis of mild cognitive impairment (aMCI) and Alzheimer’s disease (AD)), *MOCA* Montreal cognitive assessment, *CDR* Clinical dementia rating

Despite finding no significant difference between the perivascular areas of retinal venules and arterioles (Fig. 2E), the total retinal perivascular AP count was significantly higher in the peri-arteriolar (A) region compared to the peri-venular (V) region (Fig. 2F; A: 51.96 ± 19.65 vs. V: 35.57 ± 12.66, *P* < 0.0001; detailed data for each vascular subtype in Supplementary Table 2). These findings remained consistent regardless of sex (Fig. 2G-H, Supplementary Table 3) or the degree of cognitive impairment (Fig. 2I–J). Notably, the increased retinal peri-arteriolar versus peri-venular AP count was statistically more significant in individuals with impaired cognition (IC) compared to those with normal cognition (NC) (Fig. 2I versus 2J). The peri-venular AP count exceeded the peri-arteriolar plaque count (*P* = 0.013; Supplementary Table 2) only in the retinal primary main branches. Following the initial bifurcation, retinal peri-arteriolar AP burden remained significantly greater than the peri-venular AP burden at all levels, except for a similar non-significant trend observed in the tertiary branches (Supplementary Figu. 3A–E, Supplementary Table 2).

**Fig. 3:**
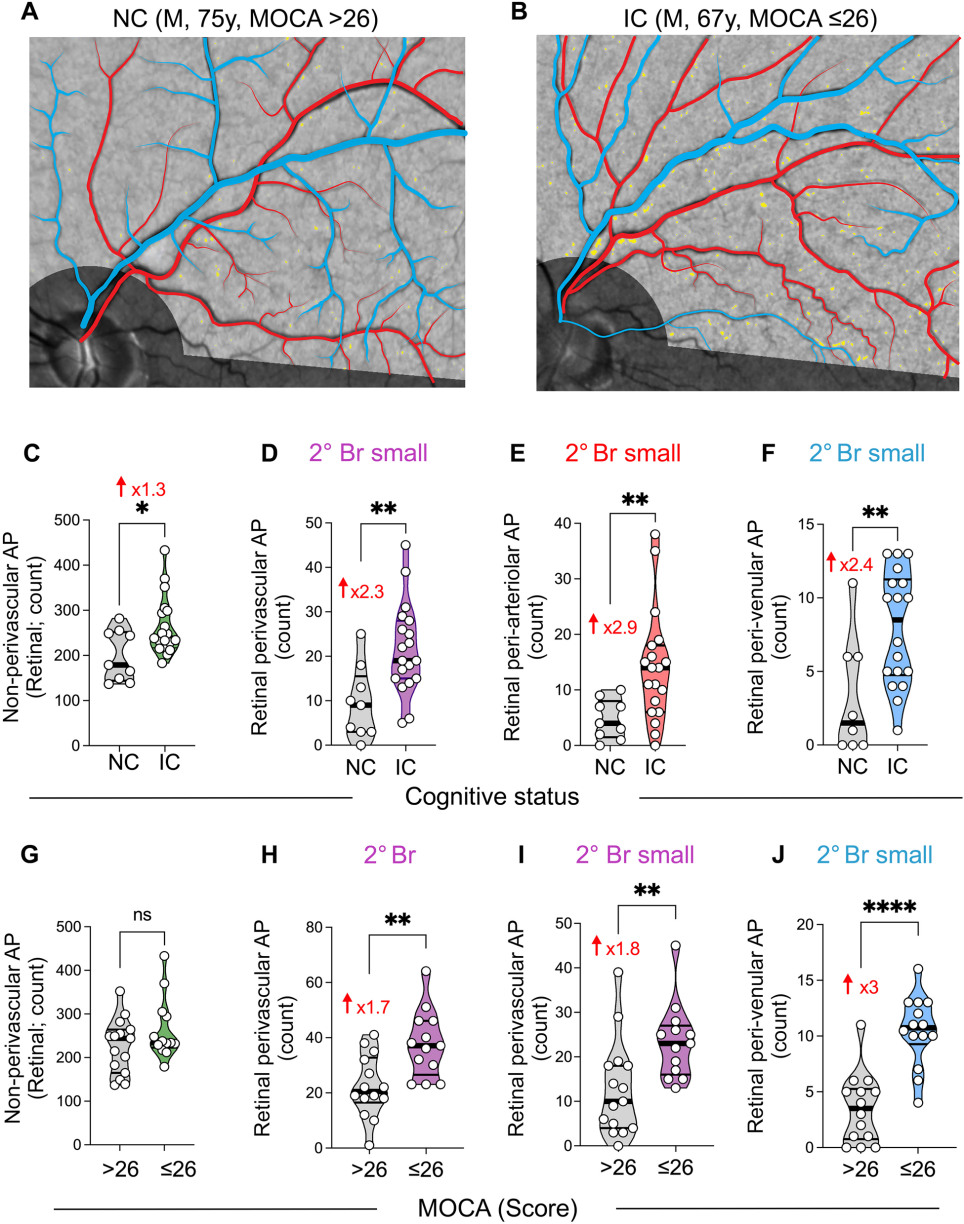
Retinal peri-arteriolar and peri-venular amyloid plaque count stratified by cognitive status and MOCA scores. **A**–**B** Representative fundus images of retinal perivascular amyloid plaque (AP) showing their density and distribution along arterioles (red tracing) and venules (blue tracing), in individuals with normal cognition (NC; **A**) or impaired cognition (IC; **B**). **C** Quantitative analyses of retinal non-perivascular AP count stratified by cognitive status, NC versus IC. **D**–**F** Quantitative analyses of retinal AP count stratified by cognitive status, in perivascular (**D**), peri-arteriolar (**E**), and peri-venular (**F**), for the secondary (2°) small branches. **G** Quantitative analyses of retinal non-perivascular AP count stratified by MOCA scores of 26 or lower compared with greater than 26. **H**–**J** Quantitative analyses of AP count stratified by MOCA, in perivascular 2° branches (**H**), 2° small branches (**I**), and peri-venular 2° small branches (**J**). Violin plots are showing individual data points, median and interquartile range. Statistics: *P < 0.05, ** *P* < 0.01, **** *P* < 0.0001, by unpaired two-tailed Student’s t test or Mann–Whitney. M, male; MOCA, Montreal Cognitive Assessment; y, years. Color code for vessel type: purple—perivascular; blue—peri-venular; red—peri-arteriolar

When stratified by cognitive status (Fig. 3A–F; Table 2; extended data in Supplementary Fig. 3F–H), IC subjects demonstrated 1.3-fold greater non-perivascular amyloid count (*P* = 0.018, Fig. 3C) and significantly greater (2.3-fold) retinal perivascular AP burden in the secondary branches compared to NC subjects (*P* = 0.0037). This difference was observed in both the total peri-venular (*P* = 0.0011) and total peri-arteriolar areas (*P* = 0.015), with a substantial increase (2.4–2.9-fold) noted in the secondary small branches (Fig. 3A–F and Table 2; extended data in Supplementary Fig. 3F–H). Tertiary branches also exhibited significantly greater retinal AP burden in the peri-venular areas in the IC group (*P* = 0.020, Supplementary Fig. 3H). Interestingly, there was a non-significant trend indicating greater perivascular AP burden in the main primary branches among NC subjects compared to IC subjects (*P* = 0.08, Table 2).

**Table 2:** Retinal perivascular amyloid plaques (AP) in cognitively normal and impaired subjects

DIAGNOSTIC GROUP		NC			IC			Fold change	Statistics
Vascular type		Mean	SD	n	Mean	SD	n	FC [95% CI]	*P* value
Perivascular AP	Total	83.89	32.04	9	89.26	27.81	19	1.1 [0.74, 1.38]	0.65
	Primary Br (total)	43.56	35.12	9	24.32	16.85	19	0.6 [0.20, 0.92]	0.20^#^
	*Primary Br—main*	12.33	7.28	9	7.32	6.60	19	0.6 [0.24, 0.94]	0.080
	*Primary Br—small*	31.22	29.55	9	17.00	11.64	19	0.5 [0.15, 0.94]	0.35^#^
	Secondary Br (total)	19.78	10.47	9	35.79	13.18	19	1.8 [1.08, 2.54]	**0.0037**
	*Secondary Br—main*	10.33	5.89	9	14.26	6.36	19	1.4 [0.77, 1.99]	0.13
	*Secondary Br—small*	9.44	8.14	9	21.53	10.15	19	2.3 [0.84, 3.72]	**0.0044**
	Tertiary Br	19.56	12.63	9	27.89	19.33	19	1.4 [0.64, 2.21]	0.24^#^
Peri- Venular AP	Total	32.11	9.40	9	37.21	13.86	19	1.2 [0.85, 1.47]	0.33
	Primary Br (total)	18.67	13.17	9	11.68	9.15	19	0.6 [0.25, 1.01]	0.11
	*Primary Br—main*	7.89	4.76	9	4.63	5.05	19	0.6 [0.20, 0.97]	0.12
	*Primary Br—small*	10.78	9.67	9	7.05	5.65	19	0.7 [0.18, 1.13]	0.21
	Secondary Br (total)	6.25	4.59	8	13.39	4.53	18	2.1 [0.45, 3.34]	**0.0011**
	*Secondary Br—main*	3.11	1.90	9	5.44	2.999	18	1.8 [0.88, 2.62]	**0.044**
	*Secondary Br—small*	3.25	4.03	8	7.94	3.90	18	2.4 [0.16, 4.73]	**0.0098**
	Tertiary Br	4.43	2.76	7	11.93	8.23	15	2.7 [1.03,4.35]	**0.020**^#^
Peri- Arteriolar AP	Total	51.78	24.70	9	52.05	17.55	19	1.0 [0.64, 1.37]	0.97
	Primary Br (total)	24.89	24.43	9	12.63	11.96	19	0.5 [0.10, 0.92]	0.24^#^
	*Primary Br—main*	4.44	4.13	9	2.68	2.77	19	0.6 [0.12, 1.09]	0.19
	*Primary Br—small*	23.00	21.27	8	10.50	10.14	18	0.5 [0.08, 0.83]	0.21^#^
	Secondary Br (total)	12.00	5.70	9	23.11	12.13	19	1.9 [1.14, 2.71]	**0.015**
	*Secondary Br—main*	7.22	4.35	9	9.11	4.43	19	1.3 [0.66, 1.96]	0.30
	*Secondary Br—small*	4.78	3.60	9	14.00	10.01	19	2.9 [1.12, 4.74]	**0.0048**^**#**^
	Tertiary Br	14.44	9.89	9	16.56	10.94	18	1.2 [0.49, 1.80]	0.63

Statistical analysis was performed using two-tailed unpaired Student’s t test or ^#^ Mann–Whitney

*AP* amyloid plaque, *Br* branch, *FC* fold change, *IC* impaired cognition, *NC* normal cognition, *SD* standard deviation

When compared to subjects with a MOCA score greater than 26 (Fig. 3G–J and Table 3; detailed data in Supplementary Fig. 3I), subjects with MOCA scores of 26 or lower exhibited a significantly greater total perivascular AP count in the secondary branches (*P* = 0.002), notably showing a substantial threefold increase in the peri-venular small branch areas (3.50 ± 3.13 vs. 10.46 ± 3.26, *P* < 0.0001). In contrast to the stratification based on cognitive status, no significant differences were noted when non-perivascular AP counts were compared between subjects with MOCA greater than 26 and those with MOCA 26 or lower (*P* = 0.15, Fig. 3G).

**Table 3:** Retinal perivascular amyloid plaques (AP) separated by MOCA score

Vascular type		MOCA > 26			MOCA ≤ 26			Fold change	Statistics
		Mean	SD	n	Mean	SD	n	FC [95% CI]	*P* value
Perivascular AP	Total	86.40	29.27	15	88.85	29.27	13	1.0 [0.76, 1.30]	0.83
	Primary Br (total)	38.60	28.06	15	21.15	18.48	13	0.6 [0.20, 0.89]	0.053^#^
	*Primary Br—main*	11.13	6.75	15	6.38	6.87	13	0.6 [0.18, 0.97]	0.077
	*Primary Br—small*	27.47	23.15	15	14.77	13.02	13	0.5 [0.18, 0.90]	0.091^#^
	Secondary Br (total)	22.43	11.24	14	37.62	12.09	13	1.7 [1.12, 2.23]	**0.002**
	*Secondary Br—main*	11.93	5.96	15	14.23	6.87	13	1.2 [0.74, 1.65]	0.35
	*Secondary Br—small*	12.67	10.71	15	23.38	8.51	13	1.8 [0.93, 2.76]	**0.0075**
	Tertiary Br	21.93	13.02	15	29.00	21.80	13	1.3 [0.62, 2.03]	0.34^#^
Peri- Venular AP	Total	32.20	10.60	15	39.46	14.09	13	1.2 [0.90, 1.55]	0.13
	Primary Br (total)	17.93	10.73	15	9.31	9.40	13	0.5 [0.18, 0.86]	**0.034**
	*Primary Br—main*	7.13	4.09	15	4.00	5.79	13	0.7 [0.07, 1.05]	0.11
	*Primary Br—small*	10.80	8.17	15	5.31	4.68	13	0.5 [0.18, 0.81]	**0.042**
	Secondary Br (total)	7.64	4.34	14	15.69	3.77	13	2.0 [1.35, 2.75]	** < 0.0001**
	*Secondary Br—main*	4.14	2.80	14	5.23	2.95	13	1.7 [0.64, 1.88]	0.33
	*Secondary Br—small*	3.50	3.13	14	10.46	3.26	13	3.0 [1.42, 4.55]	** < 0.0001**
	Tertiary Br	7.67	6.50	12	15.50	14.30	12	2.0 [0.51, 3.54]	0.10^#^
Peri- Arteriolar AP	Total	54.20	21.62	15	49.38	17.60	13	0.9 [0.64, 1.18]	0.53
	Primary Br (total)	20.67	20.70	15	11.85	11.99	13	0.8 [0.12, 1.02]	0.42^#^
	*Primary Br—main*	4.00	3.89	15	2.38	2.29	13	0.6 [0.15, 1.05]	0.20
	*Primary Br—small*	17.86	17.78	14	10.25	10.85	12	0.6 [0.09, 1.05]	0.33^#^
	Secondary Br (total)	17.47	11.94	15	21.92	11.29	13	1.3 [0.67, 1.84]	0.32
	*Secondary Br—main*	8.07	4.13	15	9.00	4.85	13	1.1 [0.66, 1.57]	0.59
	*Secondary Br—small*	9.40	10.25	15	12.92	8.50	13	1.4 [0.43, 2.32]	0.11^#^
	Tertiary Br	15.80	10.97	15	15.92	10.26	12	1.0 [0.47, 1.54]	0.98

Statistical analysis was established using two-tailed unpaired Student’s t test or ^#^ Mann–Whitney. *P < 0.05, **P < 0.01, ****P < 0.0001

*AP* amyloid plaque, *Br* branch, *FC* fold change, *MOCA* Montreal Cognitive Assessment, *SD* standard deviation

Patients with high CDR scores (Supplementary Fig. 4 and Supplementary Table 4) exhibited a greater total retinal perivascular AP count in the secondary branches (*P* = 0.020), along with significantly elevated values in the tertiary branch areas upon further topographic analysis (*P* = 0.0058; Supplementary Fig. 4A–B). Total peri-venular AP burden, particularly in the tertiary branches, was significantly greater in subjects with higher CDR scores (*P* = 0.0025 and *P* = 0.0007 respectively; Supplemental Fig. 4C–D). Moreover, both the main and small branches of peri-arteriolar secondary regions exhibited a significantly greater AP count in subjects with a CDR of 2 compared to CDRs of 0.5 and 1 (Supplementary Fig. 4E and Supplementary Table 4).

**Fig. 4:**
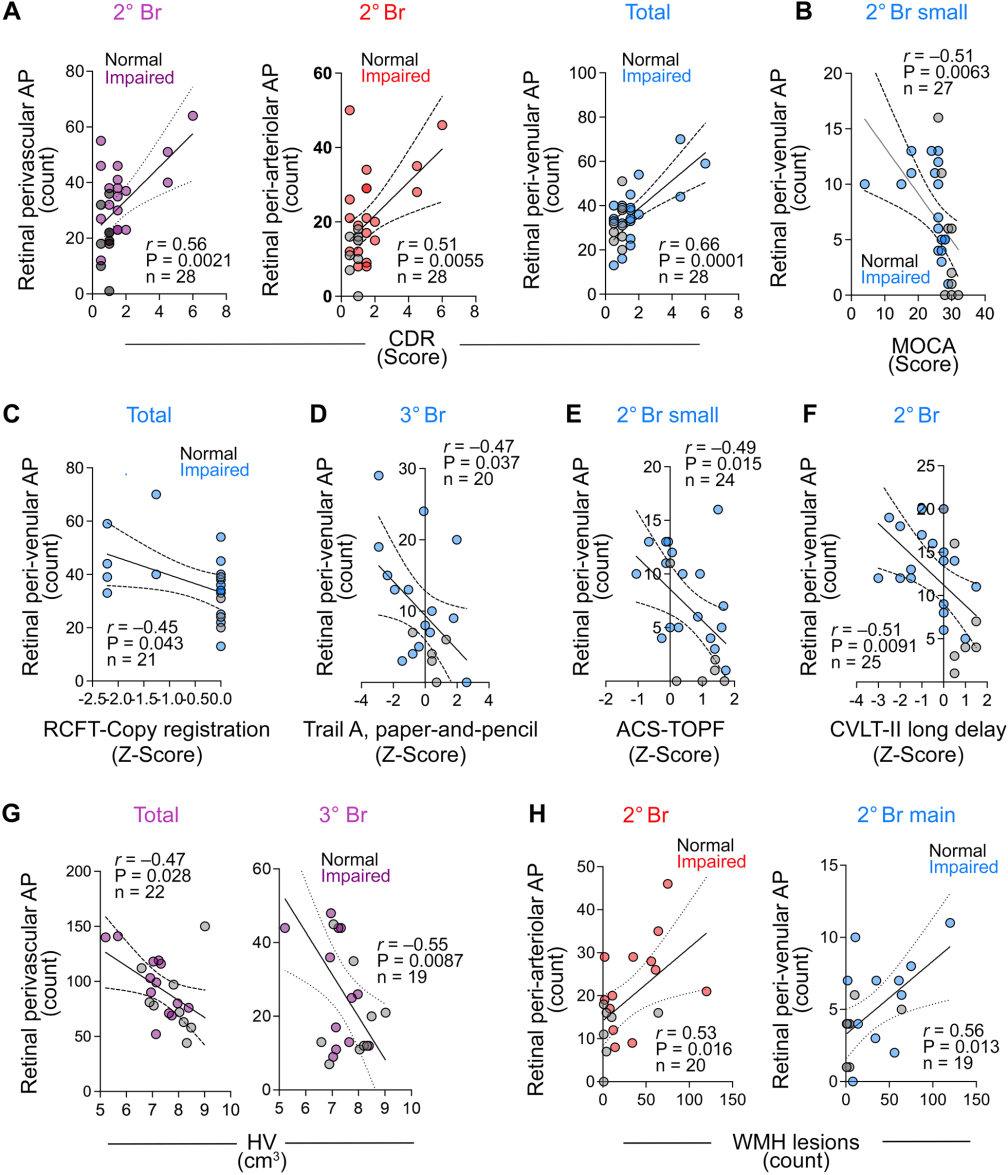
Correlations between retinal perivascular amyloid plaque distribution with cognitive and neuroimaging measures. Pearson’s *r* correlation analyses between retinal perivascular AP count and CDR (**A**), MOCA (**B**), RCFT-copy registration (**C**), Trail A-paper and pencil (**D**), ACS-TOFF (**E**), CVLT-II Long delay (**F**), hippocampal volume (**G**) and white matter hyperintensities lesions (**H**). AP, Amyloid plaques; CDR, Clinical dementia rating; MOCA, Montreal cognitive assessment; RCFT, Rey Complex Figure Test and Recognition Trial; ACS-TOPF, test of Premorbid Functioning; CVLT, California Verbal Learning Test; HV, hippocampal volume, WMH, White matter hypertensity; 2° Br, secondary branches; 3° Br, tertiary branches. Color code for vessel type: purple—perivascular; blue—peri-venular; red—peri-arteriolar

Pearson’s correlation analyses revealed a correlation between retinal perivascular AP count and CDR scores, with the most significant correlation in this cohort observed for the total peri-venular AP count (Fig. 4A and Table 4; *r* = 0.66, *P* = 0.0001). Topographically, the tertiary branch peri-venular AP count (*r* = 0.61, *P* = 0.0016) and the secondary branch peri-arteriolar AP count (*r* = 0.51, *P* = 0.0055) showed significant correlations with CDR scores (Table 4). The secondary small branch peri-venular AP count showed the highest correlation with the MOCA score (Fig. 4B; *r* = − 0.51, *P* = 0.0063).

**Table 4:** Pearson’s *r* correlations between peri-venular or peri-arteriolar AP with cognitive status and brain imaging parameters

Vascular type		MOCA n = 24–28		CDR n = 24–28		HV n = 19–22		Number of WMHI n = 17–20	
		*r*	*P*	*r*	*P*	*r*	*P*	*r*	*P*
Perivascular AP	Total	0.03	ns	0.46	0.01	− 0.47	0.028	0.34	ns
	Primary Br (total)	0.29	ns	− 0.15	ns	0.09	ns	− 0.24	ns
	*Primary Br*—*main*	0.19	ns	0.04	ns	− 0.20	ns	0.01	ns
	*Primary Br—small*	0.30	ns	− 0.21	ns	0.19	ns	− 0.31	0.045
	Secondary Br (total)	− 0.38	0.05	**0.56**	**0.0021**	− 0.41	ns	0.56	0.010
	*Secondary Br—main*	− 0.11	ns	0.40	0.034	− 0.27	ns	0.48	0.033
	*Secondary Br—small*	− 0.42	0.02	**0.49**	**0.0078**	− 0.40	ns	0.45	0.047
	Tertiary Br	− 0.07	ns	**0.53**	**0.0040**	**− 0.55**	**0.0087**	0.44	0.052
Peri- Venular AP	Total	− 0.26	ns	**0.66**	**0.0001**	− 0.51	0.016	0.40	ns
	Primary Br (total)	0.27	ns	− 0.06	ns	0.02	ns	− 0.33	ns
	*Primary Br—main*	0.20	ns	0.05	ns	− 0.15	ns	− 0.07	ns
	*Primary Br—small*	0.27	ns	− 0.12	ns	0.14	ns	− 0.45	0.045
	Secondary Br (total)	− 0.44	0.022	0.31	ns	− 0.24	ns	0.43	ns
	*Secondary Br—main*	− 0.03	ns	0.21	ns	− 0.34	ns	0.56	0.013
	*Secondary Br—small*	**− 0.51**	**0.0063**	0.25	ns	− 0.11	ns	0.15	ns
	Tertiary Br	− 0.38	ns	**0.61**	**0.0016**	− 0.51	0.026	0.52	0.032
Peri- Arteriolar AP	Total	0.21	ns	0.24	ns	− 0.37	ns	0.23	ns
	Primary Br (total)	0.25	ns	− 0.19	ns	0.12	ns	− 0.14	ns
	*Primary Br—main*	0.10	ns	0.02	ns	− 0.21	ns	0.11	ns
	*Primary Br—small*	0.25	ns	− 0.16	ns	0.06	ns	− 0.13	ns
	Secondary Br (total)	− 0.24	ns	**0.51**	**0.0055**	− 0.42	ns	0.53	0.016
	*Secondary Br—main*	− 0.13	ns	0.42	0.026	− 0.19	ns	0.31	ns
	*Secondary Br—small*	− 0.23	ns	0.43	0.022	− 0.42	0.049	0.49	0.028
	Tertiary Br	0.18	ns	0.19	ns	− 0.46	0.033	0.13	ns

Bolded values are significant after Holms-Bonferroni multiple comparisons correction

*AP* amyloid plaque, *Br* branch, *CDR* clinical dementia rating, *HV* hippocampal volume, *MOCA* Montreal Cognitive Assessment, *WMHI* White matter hyperintensities

The evaluation of specific cognitive domains (Fig. 4, Table 5; detailed data in Supplementary Fig. 5) revealed greater overall retinal peri-venular AP accumulation in subjects with lower visuo-spatial ability as assessed by the Rey Complex Figure Test and Recognition Trial (RCFT)-Copy registration (Fig. 4C; *r* = − 0.45, *P* = 0.043) and speed of information processing (Trail A paper and pencil; Table 5; *r* = − 0.42, *P* = 0.039) Z-scores. Similarly, moderate correlations were found between lower speed of information processing (Trail A paper and pencil) Z-scores and retinal AP counts in peri-venular tertiary branches (Fig. 4D; r = − 0.47, *P* = 0.037) and peri-arteriolar secondary branches (Table 5; r = − 0.44, *P* = 0.027). We also noted a higher retinal peri-venular AP count in distal secondary branches in subjects with lower Z-scores on the general cognitive Test of Premorbid Functioning ACS (Fig. 4E; r = − 0.49, *r* = 0.015) and the California Verbal Learning Test (CVLT) II Long Delay (Fig. 4F; *r* = − 0.51, *P* = 0.0091). Interestingly, positive correlations were found between peri-arteriolar primary branch AP count and RCFT recall at 20 min and CVLT-II Z-scores (*r* = 0.41, *P* = 0.039 and *r* = 0.53, *P* = 0.0052 respectively; Supplementary Fig. 5A–B). Symptom validity and functional status (SF-36PCS) also positively correlated with retinal AP count in the primary venular branches only (Supplementary Fig. 5C).

**Table 5:** Pearson’s *r* correlations between perivascular AP and cognitive domains

Vascular type		RCFT-Copy (Z Score) n = 18–21		ACS-TOPF (Z Score) n = 21–25		CVLT-II Long Delay (Z-Score) n = 22–26		Trails A paper-and-pencil (Z-score) n = 21–25	
		*r*	*P*	*r*	*P*	*r*	*P*	*r*	*P*
Perivascular AP	Total	− 0.18	ns	0.07	ns	− 0.03	ns	− 0.26	ns
	Primary Br	0.25	ns	0.20	ns	0.46	0.017	0.18	ns
	*Primary Br*—*main*	0.09	ns	0.35	ns	0.43	0.028	0.11	ns
	*Primary Br—small*	0.31	ns	0.13	ns	0.44	0.024	0.20	ns
	Secondary Br	− 0.38	ns	− 0.27	ns	− 0.41	0.036	− 0.40	0.047
	*Secondary Br—main*	− 0.31	ns	− 0.06	ns	− 0.10	ns	− 0.47	0.02
	*Secondary Br—small*	− 0.32	ns	− 0.31	ns	− 0.47	0.014	− 0.25	ns
	Tertiary Br	− 0.28	ns	0.06	ns	− 0.40	0.046	− 0.30	ns
Peri-Venular AP	Total	− 0.45	0.043	− 0.14	ns	− 0.21	ns	− 0.42	0.039
	Primary Br	0.16	ns	0.19	ns	0.28	ns	0.16	ns
	*Primary Br—main*	0.15	ns	0.29	ns	0.27	ns	0.18	ns
	*Primary Br—small*	0.14	ns	0.09	ns	0.24	ns	0.11	ns
	Secondary Br	− 0.34	ns	− 0.38	ns	**− 0.51**	**0.0091**	− 0.31	ns
	*Secondary Br– main*	0.02	ns	0.07	ns	− 0.20	ns	− 0.32	ns
	*Secondary Br—small*	− 0.44	ns	− 0.49	0.015	− 0.49	0.014	− 0.17	ns
	Tertiary Br	− 0.40	ns	− 0.17	ns	− 0.45	0.036	− 0.47	0.037
Peri-Arteriolar AP	Total	0.05	ns	0.19	ns	0.09	ns	− 0.10	ns
	Primary Br	0.24	ns	0.17	ns	0.50	0.010	0.15	ns
	*Primary Br—main*	− 0.02	ns	0.32	ns	**0.53**	**0.005**	− 0.02	ns
	*Primary Br—small*	0.24	ns	0.12	ns	0.44	0.030	0.13	ns
	Secondary Br	− 0.30	ns	− 0.12	ns	− 0.34	ns	− 0.31	ns
	*Secondary Br—main*	− 0.43	ns	− 0.10	ns	− 0.09	ns	− 0.44	0.027
	*Secondary Br—small*	− 0.17	ns	− 0.10	ns	− 0.37	ns	− 0.18	ns
	Tertiary Br	0.06	ns	0.17	ns	− 0.32	ns	− 0.05	ns

Bolded values are significant after Holms-Bonferroni multiple comparisons correction

*AP* Amyloid plaque, *Br* Branch, *RCFT* Rey Complex Figure Test and Recognition Trial, *ACS-TOPF* test of Premorbid Functioning, *CVLT* California Verbal Learning Test

Correlation analyses conducted with neuroimaging measurements revealed inverse relationships between HV and retinal AP count in the total perivascular area (Fig. 4G, left;* r* = − 0.47, *P* = 0.028), and moreover, in the tertiary branch area (Fig. 4G, right; *r* = − 0.55, *P* = 0.0087). Inverse correlations were also observed between HV and AP count in both peri-venular total and tertiary branches (*r* = − 0.51, *P* = 0.016 and *r* = − 0.51, *P* = 0.026, respectively), as well as AP count in peri-arteriolar secondary small and tertiary branches (*r* = − 0.42, *P* = 0.049 and *r* = − 0.46, *P* = 0.033, respectively; Table 4 and Supplementary Fig. 5D). In contrast, the number of WMHI (Fig. 4H, Table 4, and Supplementary Fig. 5E) positively correlated with retinal AP count in the peri-arteriolar secondary branch (*r* = 0.53, *P* = 0.016) and peri-venular secondary main and tertiary branches (*r* = 0.56, *P* = 0.013 and *r* = 0.52, *P* = 0.032 respectively). None of the perivascular AP counts exhibited significant correlations with total intracranial volumes, but some showed associations with the volume of WMHI and subcortical Fazekas scores (Supplementary Fig. 5F–G).

## Discussion

We describe the topographic distribution of retinal supero-temporal peri-arteriolar and peri-venular amyloid deposits in a noninvasive human retinal imaging study involving subjects with normal or impaired cognition (mostly amnestic MCI). To the best of our knowledge, this description has not been conducted previously. Our exploratory analysis of the topographical interaction between two retinal imaging biomarkers of AD, amyloid and vasculature, conveys novel insights into (1) the localization of perivascular retinal amyloid deposits, which, akin to AD brain patterns, were predominantly detected in the peri-arteriolar regions compared to the peri-venular regions, and (2) the relationship between the differentiated perivascular amyloidosis with neuroimaging markers of neurodegeneration and cognitive performance. We found that the secondary vascular branches have significantly higher perivascular amyloid burden in subjects with impaired cognition compared to normal cognition, irrespective of stratification by neuropsychometric diagnosis, MOCA or CDR, and this finding remained consistent across sex. Patients with even slightly lower MOCA scores and greater CDR scores had statistically significant greater peri-venular amyloid burden, which also showed significant negative correlation with HV. Moreover, secondary branch peri-arteriolar and peri-venular AP counts significantly and positively correlated with the WMHI count. These promising exploratory findings encourage future studies in larger and more diverse cohorts to assess the efficacy of monitoring AD progression, response to therapy, and the risk of amyloid-related imaging abnormalities (ARIA) through non-invasive retinal perivascular amyloid imaging.

Pericyte loss in the brain [3] and retina [16] is linked to a rapid cascade of neurodegeneration and amyloid deposition, leading to increased vascular amyloidosis in both the retina and the brain. Abnormalities in the blood-retina barrier and vascular-associated retinal Aβ deposition have been reported in postmortem retinas of AD patients, occurring inside the blood vessel walls, as well as around and along the blood vessels [11, 42, 48, 49]. More Aβ_40_ was found to be accumulated in arterioles than in venules [49]. Furthermore, various blood-retinal barrier tight junction biomarkers were found to be deficient in the retinas of MCI and AD individuals and were associated with retinal and brain amyloid burden and cognitive status [49]. Apart from the well-characterized pericyte injury, it remains unclear whether perivascular amyloidosis affects arterioles and venules differentially at various stages of AD progression [57–60].

Similar to the retina, the cerebral vasculature has been studied to understand AD pathophysiology and identify potential specific therapeutic targets [61–63]. Increased cerebral capillary damage has been shown to correlate with the level of insoluble Aβ in AD [64], and pericyte downregulation in the deep white matter has been observed in both vascular dementia and AD [65]. Aβ deposition around cerebral blood vessels, predominantly arteries (CAA), is thought to be major contributor to vascular dysfunction in AD [66–69]. The two-hit vascular hypothesis of AD emphasizes the role of neurovascular dysfunction as an early factor that favors vascular Aβ aggregation and neurodegeneration [69]: vascular dysfunction (hit one) is followed by Aβ accumulation (hit two), which precedes and promotes neurodegeneration [70]. According to the vascular hypothesis of AD, alterations in the neurovascular unit could lead to vascular Aβ accumulation, which in turn promotes neuronal dysfunction, accelerating neurodegeneration and dementia. Insoluble vascular amyloid deposits trigger neurovascular unit dysfunction in the AD brain [71]. Whereas most human studies have focused on amyloid deposition in leptomeningeal and cortical arterioles, murine models of AD have also shown abnormalities in venules [72]. Additionally, the brain’s glymphatic system is impaired in AD, leading to insufficient amyloid clearance [73], hence the development of novel perivascular clearance system imaging techniques are underway [74].

Since the phenomenon of vascular amyloid clearance potentially leading to perivascular amyloidosis had not been inspected in the retina, we conducted this topographic analysis of retinal fluorescent images to assess the distribution of AP in relation to the primary, secondary, and tertiary retinal arteriolar and venular branches in subjects with normal and impaired cognition. In the supero-temporal quadrant of the retina, we noted a higher prevalence of perivascular amyloidosis in the peri-arteriolar region compared to the peri-venular region, both overall and across primary, secondary, and tertiary branches. Our discovery that vascular amyloid accumulation occurs more frequently along the arterioles in the retina is consistent with patterns observed in cerebral arterial amyloidosis [16, 68, 69]. We also noted a significant increase in retinal amyloidosis in the peri-venular and peri-arteriolar secondary and tertiary branch regions in subjects with CDR scores of 1 and 2, as well as MOCA scores lower than 26. Additionally, the perivascular amyloid burden around the secondary small and tertiary branches inversely correlated with HV. The secondary branch peri-arteriolar and peri-venular plaque burden positively correlated with the number of WMHI lesions.

These findings generate further hypotheses. Venular narrowing and increased venular tortuosity were described to correlate with the cognitive status and AP burden in AD [15, 17, 26, 29, 75]. It is conceivable that the overall retinal peri-venular AP burden could emerge as a marker of cognitive impairment, while the burden of perivascular AP in secondary branches may correlate of neurodegeneration markers, such as hippocampal atrophy and WMHI burden. This suggests that although peri-arteriolar amyloid deposition may occur earlier, peri-venular amyloid accumulation becomes more apparent later in the disease process and could serve as an indicator of more advanced neurodegenerative disease progression. The association between WMHI and CAA is gaining increased recognition [76]. The deposition of Aβ_40_ in the walls of cerebral arteries suggests an age-related failure of perivascular drainage of soluble Aβ from the brain. White matter pathology is proposed to be the link between blood–brain barrier leakage and the decline in information processing speed in older individuals, with and without cognitive impairment [77]. The negative association between white matter blood–brain barrier leakage and information processing speed performance was mediated by the WMHI volume, with no correlation observed with HV [77]. Disruption of the brain microcirculation not only contributes to amyloidopathy but also initiates a non-amyloidogenic pathway of vascular-mediated neuronal dysfunction and injury, characterized by increased permeability of blood vessels, leakage of blood-borne components into the brain, and, consequently, neurotoxicity. The diminished brain capillary flow leads to multiple focal ischemic or hypoxic microinjuries, diminished Aβ clearance, and the formation of neurotoxic oligomers, ultimately resulting in neuronal dysfunction [78].

There appears to be a greater prevalence of retinal amyloidosis around first-order branches in cognitively intact individuals, while significantly greater retinal amyloidosis is found around smaller branches in patients with impaired cognition. The reduced complexity of the retinal vascular branching network, as previously described in subjects with MCI and AD [15, 26, 29, 79], may explain the preferential accumulation of perivascular amyloid in secondary branches in cognitively impaired individuals. Interestingly, an agnostic machine learning-based study identified the same secondary branch perivascular area on heat-maps derived from retinal photographs, discriminating between AD individuals and healthy controls with high accuracy [80].

Our results within small subgroups should be interpreted cautiously and require confirmation in larger well-powered studies, further exploring the clinical utility of perivascular amyloidosis topography and the underlying pathophysiology of peri-arteriolar and peri-venular amyloid deposition at various stages of AD progression. With this current methodology, we were unable to assess the retinal far periphery or determine the status of amyloid burden adjacent to capillaries, both of which warrant future investigation. Furthermore, automated protocols to analyze retinal perivascular amyloidosis in the whole fundus are needed. Other study limitations include the relatively small number of subjects with cognitive decline, predominantly of Caucasian race, and the lack of AD biomarker confirmation, which reduces the generalizability of our findings.

## Conclusions

In this exploratory study of the topographical interaction between retinal vasculature and Aβ deposits, we discovered that increased supero-temporal retinal Aβ deposits in the distal peri-venular regions can differentiate between normal and impaired cognition and are inversely associated with hippocampal neurodegeneration. Our findings from both in vivo imaging and histopathological analyses reveal similar patterns of perivascular Aβ depositions. These data further support the hypothesis that the pathophysiology of AD in the retina and brain is analogous, evidenced by the preferential accumulation of amyloid in the peri-arterial regions. However, the relationship between retinal perivascular amyloidosis and cognitive performance is complex and necessitates further investigation through longitudinal prospective studies involving a larger and more diverse cohort with confirmation of cerebral amyloid status via amyloid-PET imaging. It is worthwhile to further explore and validate the potential of peri-arteriolar and peri-venular amyloid burden as biomarkers across various stages of AD and as potential risk for ARIA.

### Supplementary Information

Additional file1.

## Data Availability

The datasets used and/or analyzed during the current study available from the corresponding author on reasonable request.

## Abbreviations

AD: Alzheimer’s disease
Aβ: Amyloid β-protein
MCI: Mild cognitive impairment
SLO: Scanning laser ophthalmoscopy
AP: Aβ plaques
CAA: Cerebral amyloid angiopathy
IRB: Institutional Review Board
MOCA: Montreal Cognitive Assessment
CDR: Clinical Dementia Rating
(WAIS)-IV: Wechsler Adult Intelligence Scale
CVLT: California Verbal Learning Test
WMS: Wechsler Memory Scale
RCFT: Rey Complex Figure Test
BVMT-R: Brief Visuo-Spatial Memory Test Revised
RCFT: Rey Complex Figure Test
PCS: Physical Component Score
MCS: Mental Component Score
MRI: Magnetic resonance imaging
ICV: Intracranial volume
HV: Hippocampal volume
ILVV: Inferior lateral ventricle volume
WHMI: White matter hyperintensities
ANOVA: One-way analysis of variance
IC: Impaired cognition
NC: Normal cognition
